# Invariance of visual operations at the level of receptive fields

**DOI:** 10.1186/1471-2202-14-S1-P242

**Published:** 2013-07-08

**Authors:** Tony Lindeberg

**Affiliations:** 1 Department of Computational Biology, School of Computer Science and Communication, KTH Royal Institute of Technology, SE-100 44 Stockholm, Sweden

## 

Receptive field profiles measured by cell recordings have shown that mammalian vision has developed receptive fields tuned to different sizes and orientations in the image domain as well as to different image velocities in space-time [1,2]. This article presents a theory by which families of *idealized receptive field profiles *can be derived mathematically from a small set of basic assumptions that correspond to structural properties of the environment [3,4]. The article also presents a theory for how basic invariance properties to variations in scale, viewing direction and relative motion can be obtained from the output of such receptive fields, using complementary selection mechanisms that operate over the output of families of receptive fields tuned to different parameters [4]. Thereby, the theory shows how basic invariance properties of a visual system can be obtained already at the level of receptive fields, and we can *explain *the different shapes of receptive field profiles found in biological vision from a requirement that the visual system should be invariant to the natural types of image transformations that occur in its environment.

## Model

The brain is able to maintain a stable perception although the visual stimuli vary substantially on the retina due to geometric transformations and lightning variations in the environment. These transformations comprise (i) local scaling transformations caused by objects of different size and at different distances to the observer, (ii) locally linearized image deformations caused by variations in the viewing direction in relation to the object, (iii) locally linearized relative motions between the object and the observer and (iv) local multiplicative intensity transformations caused by illumination variations. Let us assume that receptive fields should be constructed by linear operations that are shift-invariant over space and/or space-time, with an additional requirement that receptive fields must not create new image structures at coarser scales that do not correspond to simplifications of corresponding structures at finer scales.

## Results

Given the above structural conditions, we derive idealized families of spatial and spatio-temporal receptive fields that satisfy these structural requirements *by necessity*, based on Gaussian kernels, Gaussian derivatives or closely related operators [3,4]. We show that there are very close similarities between the receptive fields predicted from this theory and receptive fields found by cell recordings in biological vision, including (i) spatial on-center-off-surround and off-center-on-surround receptive fields in the fovea and the LGN, (ii) simple cells with spatial directional preference in V1, (iii) space-time separable spatio-temporal receptive fields in the LGN and V1 and (iv) non-separable space-time tilted receptive fields in V1 [3,4]. Indeed, from kernels predicted by this theory it is possible to generate receptive fields similar to all the basic types of monocular receptive fields reported by DeAngelis et al [2] in their survey of classical receptive fields.

By complementing such receptive field measurements with selection mechanisms over the parameters in the receptive field families, we show how *true invariance *of receptive field responses can be obtained under scaling transformations, affine transformations and Galilean transformations [4]. Thereby, the framework provides a mathematically well-founded and biologically plausible model for how basic invariance properties can be achieved already at the level of receptive fields. In this way, the presented theory supports invariant recognition of objects and events under variations in viewpoint, retinal size, object motion and illumination.
